# Novel Feature-Extraction Methods for the Estimation of Above-Ground Biomass in Rice Crops

**DOI:** 10.3390/s21134369

**Published:** 2021-06-25

**Authors:** David Alejandro Jimenez-Sierra, Edgar Steven Correa, Hernán Darío Benítez-Restrepo, Francisco Carlos Calderon, Ivan Fernando Mondragon, Julian D. Colorado

**Affiliations:** 1 Department of Electronics and Computer Science, Pontificia Universidad Javeriana Cali, Cali 760031, Colombia; davidjimenez@javerianacali.edu.co (D.A.J.-S.); hbenitez@javerianacali.edu.co (H.D.B.-R.); 2 School of Engineering, Pontificia Universidad Javeriana Bogota, Cra. 7 No. 40-62, Bogota 110311, Colombia; e_correa@javeriana.edu.co (E.S.C.); calderonf@javeriana.edu.co (F.C.C.); imondragon@javeriana.edu.co (I.F.M.)

**Keywords:** data-fusion, feature-extraction, multispectral imagery, crop biomass, phenotyping

## Abstract

Traditional methods to measure spatio-temporal variations in above-ground biomass dynamics (AGBD) predominantly rely on the extraction of several vegetation-index features highly associated with AGBD variations through the phenological crop cycle. This work presents a comprehensive comparison between two different approaches for feature extraction for non-destructive biomass estimation using aerial multispectral imagery. The first method is called GFKuts, an approach that optimally labels the plot canopy based on a Gaussian mixture model, a Montecarlo-based K-means, and a guided image filtering for the extraction of canopy vegetation indices associated with biomass yield. The second method is based on a Graph-Based Data Fusion (GBF) approach that does not depend on calculating vegetation-index image reflectances. Both methods are experimentally tested and compared through rice growth stages: vegetative, reproductive, and ripening. Biomass estimation correlations are calculated and compared against an assembled ground-truth biomass measurements taken by destructive sampling. The proposed GBF-Sm-Bs approach outperformed competing methods by obtaining biomass estimation correlation of 0.995 with $R^{2}=0.991$ and RMSE=45.358 g. This result increases the precision in the biomass estimation by around 62.43% compared to previous works.

## 1. Introduction

Rice is an essential grain to ensure global food security [1], contributing to 20% of food energy requirements worldwide. Major efforts have emerged to develop novel high-throughput methods for enabling precision biomass characterization aimed at improving crop yield, grain quality and crop management [2,3,4].

Monitoring rice biomass at larger crop scales requires remote sensing approaches for precision sampling, mostly based on unmanned aerial vehicle (UAV-driven) multispectral imagery [3,4,5,6]. For this purpose, Above-Ground Biomass Estimation (AGBE) methods have recently gained significant traction [7,8,9,10], since machine learning models can be trained by features extracted according to different plant reflectance wavelengths, namely vegetation indices (VIs). These VIs, are computed from multispectral imagery captured by UAVs or satellites. Although the combination of several VIs tends to avoid saturation issues with higher values of accumulated biomass [11], in some cases the estimation of the biomass (based on VI extracted features) requires independent models that must be characterized and calibrated dependent on the crop growth stages. Furthermore, VI features are highly affected by the genotype (rice variety) and external abiotic conditions [5,6,12,13,14].

Most of the AGBE methods reported in the literature require image segmentation algorithms to extract the VI formulas from the corresponding canopy [3,4,5,6,15]. Therefore, the accuracy of the extracted VI-features strictly depends on the quality of the segmentation, where several factors such as image resolution, saturation and radiation noise, play an important role in achieving an accurate correlation between the segmented pixels (VI-features) with the biomass. To overcome this problem, recent approaches based on graphs [4] propose the fusion of different wavelengths images into a single graph, where the eigenvectors can be used as features. As a result, graph-based methods do not require image segmentation or other photogrammetry corrections that can be computational expensive.

In this paper, we compare two approaches for the extraction of relevant features from multispectral aerial imagery, allowing the estimation of above-ground biomass based on training classical machine learning models. In previous work reported in [3,4], we introduced the aforementioned methods. The former method is called GFKuts, which was primarily used as an image segmentation method to optimally segment the crop canopy. The latter was based on graphs data fusion, which instead of using VIs or a mask image to extract features, it use the structural information captured by the graphs.

## 2. Related Work

Several approaches are used to address the above-ground biomass estimation (AGBE) problem [3,4,5,6,15]. For instance, authors in [15] conducted a comprehensive survey to identify which VIs were suitable for estimating rice biomass as a function of the growth stages of the crop. Furthermore, in [15,16], crop features were extracted by using classical k-means binary clustering for segmentation, where linear multi-variable regression models were applied to each crop stage independently for the biomass characterization. In [3], a more sophisticated method named GFKuts was presented, aimed at combining a Monte Carlo K-means with the well-known GrabCut segmentation method [17,18]. Overall, the GFKuts approach is based on three image processing methods: Magic Wand, Intelligent Scissors [19,20], and GrabCut that is grounded on the Graph-Cut method [17,18]. In GFKuts, the energy function of the Graph-Cut method was adjusted, to achieve a global optimization for a N-dimensional image through a Gaussian Mixture Model. Also, the proposed GFKuts algorithm is fully automatic [21,22].

In [23], a deep learning approach was used for the estimation of biomass in forage grass crops. Convolutional neural networks were trained with extensive datasets that are computational expensive to process, while over-fitting issues were also observed. Other approaches based on structural information combine multiple feature sources through a fused graph [4,24,25]. These methods relies on the approximation of the graphs given by the Nyström extension. In this paper, we extend the original graph method presented in [4], by adding a new sampling technique known as blue-noise sampling. This is combined with a graph signal processing approach to enhance the estimation given by the Nyström extension of the graph in [4]. As mentioned, this method uses the eigenvector from the graphs as the features to extract from the multispectral images. As a result, we expect to improve on the estimation of the above-ground biomass, by correlating the estimations against biomass measurements directly obtained from the crop by destructive sampling.

## 3. Materials and Methods

**Figure 1:**
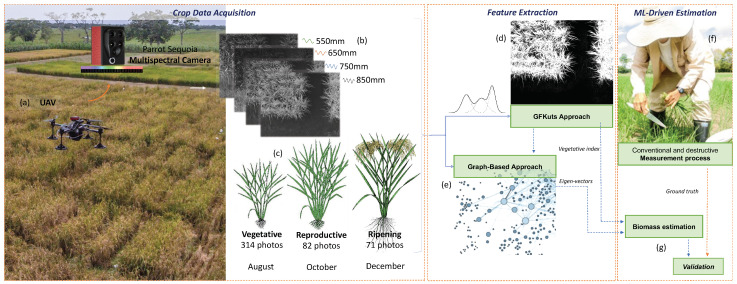
(**a**) UAV-driven remote sensing of above-ground biomass in rice crops. (**b**) Multispectral imagery. (**c**) Dataset amount & crop stages (**d**) First methodology—GFKuts. (**e**) Second methodology—Graph based. (**f**) Destructive biomass sampling. (**g**) Validation and correlation stage.

The general architecture for UAV-driven remote sensing of above-ground biomass in rice crops is presented in Figure 1. An UAV is equipped with a multispectral camera onboard that captures the reflectance of the light spectrum in four different bands (Green, Red, Red-Edge, and NIR). A set of 1868 (i.e., one image per band) images was acquired for the crop’s three growth stages: vegetative, reproductive, and ripening. Further details on the dataset acquisition and crop characteristics can be found in [3].

As mentioned, this paper presents two approaches for feature extraction. The first approach is GFKuts, which is based on the image processing area. This approach is an entirely automatic proposal that integrates a binary classification technique with an optimization approach based on a Gaussian mixture model, followed by a filtering stage, to extract a mask related to pixels that belong to the crop canopy. The second approach is a graph-based framework that aims to fuse data by taking advantage of the structural relationship between the data captured by independent graphs (i.e., the structural relationship of the pixels of an image with one graph per image channel). The method generates a resultant fused graph with the most relevant information. Unlike GFkuts, this method uses eigenvectors of the fused-graph as features rather than vegetation indices. Lastly, the extracted features obtained from both methods are the input of a regression model to estimate the above-ground biomass. The next subsections explain the features extraction approaches and the regressors.

### 3.1. GFKuts

**Figure 2:**
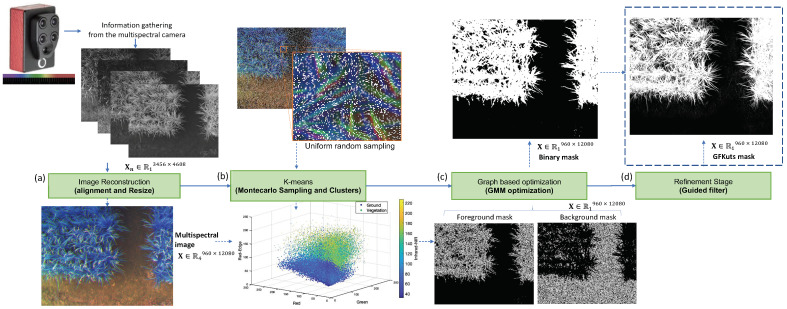
GFKuts approach. (**a**) Preprocessing stage, (**b**) binary classification approach, (**c**) GMM modeling & optimization, and (**d**) filter stage.

Figure 2 details the proposed GFKuts approach. The first contribution of GFKuts is to apply a random sampling approach based on Monte Carlo method and integrate these samples into a binary classification method [26]. This random arrangement and the binary classification algorithm solve a time-demanding computational iteration problem as they provide an efficient stochastic numerical method based on a photogrammetric image technique used in precision agriculture.

An array $I=(i_{1},\ldots ,i_{n})$ of size *N*, denotes the input image in the standard RGB color space (sRGB). The binary non-supervised classification method implemented in this work is the k-means clustering algorithm that cluster the set of *N* samples into *K* groups. The semantic labeling of the groups is random according to the nature of the algorithm. Thus, there is no control of the true class of the label assigned. Given that the results are highly repeatable, the identification of each group is carried out through the characterization of the centroid. This characterization runs only once and operates as long as the conditions of the images do not change (e.g., the type of crop, growing, or environmental conditions).

The Montecarlo sample K-means classification strategy generates a selection of pixels with a uniform random distribution of the spatial coordinates $n_{\left(x,y\right)}$ of the image. The result is a subset of sRGB values for each selected pixel *N*. These values come from the refractive bands of the light spectrum captured by the sensor. The implementation of this first stage is shown in Algorithm 1. The result is a pair of masks that allow us to initialize regions of the image to implement the optimization process.

**Algorithm 1:** Montecarlo Sampled K-means. **Input:** the image I, the number of samples *N*, and two heuristic values associated with the mean expected  radiance of the canopy: $C_{1}$, and the ground: $C_{2}$.  **for** Each pixel in range (1 *…*
*n*) **do**   Random (*x*, *y*) pixel selection from I to *P*   Store sRGB value from *P* to ${\mathrm{Feature}}_{n}$   Store pixel coordinates $I_{x,y}$  **end for**  Run K-means over Feature to get labeling $I_{n,k=1}$ and $I_{n,k=2}$  calculate the Euclidean distance between *C*_1_ and the centroid of the group *K* = 1 to m1;  calculate the Euclidean distance between *C*_1_ and the centroid of the group *K* = 2 to m2;  **if**
m1 < m2
**then**   group *K* = 1 to canopy   group *K* = 2 to ground  **else**   group *K* = 1 to ground   group *K* = 2 to canopy  **end if**  Create a mask $T_{F}$ and set the coordinates in $I_{x,y}$ of each pixel in $I_{n,canopy}$ as the foreground.  Create a mask $T_{B}$ and set the coordinates in $I_{x,y}$ of each pixel in $I_{n,ground}$ as the background.

The image segmentation consists of inferring the unknown opacity variables, denoted as α, from the given image data *I* and the model θ [18]. The opacity term $\alpha =(\alpha_{1},\ldots ,\alpha_{N})$ is the image segmentation weighed by each pixel with $0\leq \alpha_{N}\leq 1$ for soft segmentation and $\alpha_{N}\in \left(0,1\right)$ for hard segmentation, with 1 denoted as foreground and 0 for background. The parameters θ describe image foreground and background distributions modeled by GMMs values, that introduces the covariance parameter $k=(k_{1},\ldots ,k_{N})$ with $k_{n}\in \left(1,\ldots ,K\right)$ assigned for each pixel. The “Gibbs” energy function of Equation (1) denoted as E, models a trend of solidity of the objects through the opacity parameter, whose minimum corresponds to an optimal segmentation.

(1) $$E\left(\underline{\alpha },k,\underline{\theta },I\right)=U\left(\underline{\alpha },k,\underline{\theta },I\right)+V\left(\underline{\alpha },I\right).$$

The function U() in Equation (1) is explained in Equation (2); this function estimate the fitness of the opacity distribution α of the image, given the model θ, in which p() is a Gaussian probability distribution and π(·) are mixed weights coefficients.

(2) $$U\left(\underline{\alpha },k,\underline{\theta },I\right)=\sum_{n}-logp\left(z_{n}|\alpha_{n},k_{n},\theta \right)-log\pi \left(\alpha_{n},k_{n}\right)$$

The remaining function V() shown in Equation (3) is called the smoothness term. The constant γ is defined empirically as 50 by [18], given images in a 0–255 range. β is a constant that regulates the smoothness term.

(3) $$V\left(\underline{\alpha },z\right)=\gamma \sum_{\left(m,n\right)}{dis(m,n)}^{-1}\left[\alpha_{m}\neq \alpha_{n}\right]\beta *exp{\left(Z_{m}-Z_{n}\right)}^{2}$$

The segmentation can be estimated as a global minimum of α parameter over the energy model, as seen in Equation (4)

(4) $$\alpha^{\prime }=\underset{\alpha }{argminE}\left(\underline{\alpha ,\theta }\right)$$

After the optimization process, an image filtering stage is introduced. Linear and time-invariant filters are usually used. A powerful approach, is to optimize a quadratic function that directly imposes constraints on the unknown output. The solution is obtained by solving a dispersion matrix encoded with the information from the guide image [27,28]. Processing is done around each pixel using a weighted average of the nearby pixels. The processing information is based on the color and intensity of the guide image [27]. This proposal is developed with the bilateral filter, starting with the guided filter. The filter can preserve the edges and smooth out small fluctuations [29]. This approach is based on explicitly constructing the cores using an orientation image. The output is a linear transform of the guide image. This filter has the edge-preserving and anti-aliasing property such as a bilateral filter, but does not suffer from gradient inversion problems [28,29].

The filter stage has two inputs, a guide image ‘*I*’ and an input image ‘*p*’. The output is expressed as a weighted average ‘*q*’. The parameters *i* and *j* are the pixel indices, as seen in Equation (5)

(5) $$q_{i}=\sum_{j}W_{ij}\left(I\right)p_{j}$$

The guide image ‘*I*’ is the reference of the filter kernel $W_{ij}$ independent of ‘*p*’. The kernel weights can be explicitly expressed by Equation (6)

(6) $$W_{ij}^{GF}\left(I\right)=\frac{1}{{\left|\omega \right|}^{2}}\sum_{k;\left(i,j\right)\in \omega_{k}}\left(1+\frac{\left(I_{i}-\mu_{k}\right)\left(I_{j}-\mu_{k}\right)}{\sigma_{k}^{2}+\epsilon }\right)$$

The parameters $\mu_{k}$ and $\sigma_{k}^{2}$ are the mean and variance of $w_{k}$ in image ‘*I*’ respectively, ϵ is a regularization parameter and ∥w∥ is the number of pixels in $w_{k}$ [28].

### 3.2. Graph-Based Data Fusion

Multi-channel input images denoted as $X_{n}^{c}\in R^{m\times n},$ with $\;c=1,\cdots ,C_{h}$ and n=1,⋯,N, where $C_{h}$ is the number of channels of each image and *N* is the total number of images. Here, the goal is to extract relevant features given by each channel of the images, in order to train a model that captures the biomass behavior across the crop stages. As explained in [4], each channel of images are represented by a corresponding graph, with the purpose of obtaining a single fused-graph from all the channels. The fused-graph is then used as an embedded space of images to extract relevant features.

The graphs used in the approach in [4] are undirected, and they are denoted as a triplet G=(V,E,w), consisting of vertexes V, and edges E⊂V×V, and a non-negative weight function w:V×V↦[0,∞). Therefore, a multichannel image $X_{n}^{c}$ can be represented as graph G by defining a vertex set V, an edge set E⊂V×V, where each edge represents the relationship between two pixels, and a weight function *w* that measures the strength of that relationship. Typically *w* is defined by:

(7) $$\begin{array}{c} w\left(v_{i},v_{j}\right)=\exp\left(\frac{-\mathrm{dist}{\left(v_{i},v_{j}\right)}^{2}}{\sigma^{2}}\right), \end{array}$$

where $\mathrm{dist}(v_{i},v_{j})$ is the Euclidean distance between the nodes $v_{i}$ and $v_{j}$ associated to the pixels $\in X_{n}^{c}$, and σ>0 is a scaling parameter.

Given the graphs for all channels in the image ($X_{n}^{c}$) $G^{c}=\left(V,E^{c},w^{c}\right)\in R^{mn\times mn}$, a fused graph $G^{f}=\left(V,E^{f},w^{f}\right)\in R^{mn\times mn}$ can be defined by combining the weight functions of $G^{c}$ as follows:

(8) $$\begin{array}{c} w^{f}\left(v_{i},v_{j}\right)=\min\left(w^{1}\left(v_{i},v_{j}\right),w^{2}\left(v_{i},v_{j}\right),\cdots ,w^{C_{h}}\left(v_{i},v_{j}\right)\right), \end{array}$$

Therefore, the eigenvectors (u) and eigenvalues (λ) are computed from $W^{f}\in R^{mn\times mn}$ by solving the eigen problem of the normalized Laplacian, defined as:

(9) $$\begin{array}{c} \mathrm{Lu}=\lambda \mathrm{Du}, \end{array}$$

where the Laplacian $L=I-D^{-1/2}W^{f}D^{-1/2}$ and D is the degree matrix that is diagonal has elements $d_{j}=\sum_{i}w^{f}\left(v_{i},v_{j}\right)$).

#### 3.2.1. Nyström Extension

Since the matrix $W^{f}\in R^{P\times P}$ with P=mn cannot be computed the authors in [4] leveraged the Nyström extension to compute the eigenvectors of $W^{f}$ without computing the complete matrix, while using some samples ($n_{s}$) of $X_{n}^{c}$, as follows:

$$W^{f}=\left[\begin{array}{cc} A & B\\ B^{⊤} & C \end{array}\right],$$

where $A\in R^{n_{s}\times n_{s}}$, $B\in R^{n_{s}\times \left(P-n_{s}\right)}$ and $C\in R^{\left(P-n_{s}\right)\times \left(P-n_{s}\right)}$. This method approximates C by using $n_{s}$ samples from the total number of pixels $P\in X_{n}^{c}$ with $n_{s}\ll P$. Thus, the eigenvectors of the matrix $W^{f}$ can be spanned by eigenvalues and eigenvectors of A, by solving the diagonalization of A ($A=U^{⊤}\lambda U$). Hence, the eigenvectors of $W^{f}$ can be spanned by $\hat{U}={\left[\begin{array}{c} U;B^{⊤}U\lambda^{-1} \end{array}\right]}^{⊤}$. Since the approximated eigenvectors $\hat{U}$ are not orthogonal, as explained in [30], they can be obtained with $S=A+A^{-\frac{1}{2}}BB^{⊤}A^{-\frac{1}{2}}$. Then, by the diagonalization of S ($S=U_{s}\lambda_{s}U_{s}$) the final approximated eigenvectors of W are given by:

(10) $$\hat{U}=\left[\begin{array}{c} A\\ B^{⊤}A^{-\frac{1}{2}} \end{array}\right]U_{s}\lambda_{s}^{-\frac{1}{2}}.$$

Up to this point we have discussed the approach proposed by authors in [4] in which, they used the common Gaussian kernel-based graph that might not provide the best representation of the intrinsic behavior of the data. In addition, the uniformly spaced distributed sampling method used to extract samples for the Nyström extension can overlook relevant samples that contain structural information about the change detection. Moreover, as defined by [31], the approximation of $W^{f}$ given by the Nyström extension is highly affected by the way samples are selected. Thus, we propose to use a recent graph-based sampling method named as Blue noise sampling [25] alongside a graph using prior smoothness learning, which is based on graph signal representation [24]. This is detailed in the following.

#### 3.2.2. Graph Signal Processing: Smoothness Prior

The problem of learning a graph with a prior of smoothness, is to learn the Laplacian matrix (L), so that the signal variation on the resulting graph (Q(L)), is small. Here a small value of Q(L), means that the signal on the graph takes similar values to its neighbors, resulting in edge disconnections from the graph [32]. The measure of smoothness of a signal *x* on a graph is given by:

$$Q\left(L\right)=\frac{1}{2}\sum_{i,j}w_{ij}{\left(x\left(i\right)-x\left(j\right)\right)}^{2},$$

where $w_{ij}$ is the ijth entry of matrix W. To do so, we use the recent approach in [24] for large scale graphs with prior smoothness, where the authors leverage the desired graph sparsity to reduce computational cost. This model needs as inputs the number of neighbors *K* that are related to the edges per node, and the parameters α and β. Therefore, the minimization problem of [24] can be computed as:

(11) $$\begin{array}{cc} \min_{W\in R^{P\times P}} & \;\mathrm{tr}\left(W^{T}Z\right)-\alpha \sum_{i}\log\left(\sum_{j}w_{ij}\right)\\ \; & \;+\frac{\beta }{2}{∥W∥}_{F}^{2}+\frac{c}{2}{∥W-W_{0}∥}_{F}^{2}\\ s.t. & \;w_{ij}=w_{ji}\geq 0,\;i\neq j,\;w_{ij}=0,\;i=j \end{array}$$

where Z is a pairwise distances matrix, α is a log prior constant (>α→> weights in W), β is a ${∥W∥}_{F}^{2}$ prior constant (>β→ less sparsity in W), and the parameter *c* encourages adjacency matrices close to a pre-specified adjacency matrix $W_{0}$. To reduce the complexity of Equation (11) the authors in [24] fix the parameters α=β=1 and multiply the pairwise distances matrix (Z) by a parameter θ to make the sparsity analysis simpler. The parameter θ is computed by each column b of Z, and it has upper and lower bounds, as described by Equation (12).

(12) $$\begin{array}{cc} \theta_{lower} & =\sum_{j=1}^{n}\frac{1}{n\sqrt{Kz_{K+1,j}^{2}-b_{k,j}z_{K+1,j}}}\\ \theta_{upper} & =\sum_{j=1}^{n}\frac{1}{n\sqrt{Kz_{K,j}^{2}-b_{k,j}z_{k,j}}} \end{array}$$

The process is summarized in Algorithm 2, as follows:
**Algorithm 2:** Graph learning with prior smoothness [24]. **Input**: Matrix of distances *Z*, *K* edges per node (sparsity level) **Output**: Graph learned with prior smoothness $W_{s}$ **Step to compute θ**  Compute bounds of θ with Equation (12).  Compute θ as a geometric mean between the bounds $\theta_{lower}$ and $\theta_{upper}$. **Step to compute adjacency matrix $W_{s}$.**  Compute $W_{s}$ from Equation (11) with Z=θZ

#### 3.2.3. Blue-Noise Sampling

Many methods to perform a sampling over graphs can be found in the literature [33] in order to find a suitable sampling set S⊂V∈G. However, as explained in [34], is desirable to get samples from different structures or objects that are present in the image, that from the graph perspective this structures/objects can be capture by sub-graphs. Therefore, we will focus on the sampling method known as blue-noise sampling since it focus on extracting samples related to sub-graphs. This kind of sampling has been widely used in digital half-toning [35], but has recently been extended to graphs [25].

The blue-noise sampling gives as a result a subset S of vertices V that are as far as possible from each other in terms of the geodesic distances on G. The work in [25] demonstrated that the final subset given by blue-noise sampling leads to an accurate reconstruction of signals that is energy is mostly concentrated at the lowest eigenvectors of the graph Laplacian of G.

**Figure 3:**
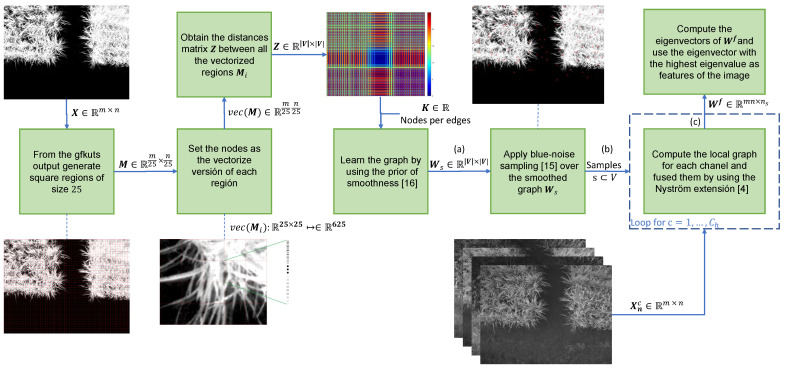
Proposed method based on the three stages: (**a**) Graph learning with prior smoothness, (**b**) blue-noise sampling to inject the samples to Nyström extension and (**c**) the fusion of the the multispectral images to extract features of the crop.

In order to assemble all the aforementioned methods into a single approach, we need a graph that contains the most relevant samples (i.e., pixels). To do this, we use a down-sampled version of the image taken from the GFKuts method [3] which contains probabilities of the pixels that belong or do not belong to the crop (see Figure 3). The down-sampling is conducted to avoid computer memory saturation, by applying a square grid $M\in R^{\frac{m}{25}\times \frac{n}{25}}$. Secondly, to learn a graph by using the Algorithm 2 of the prior of smoothness, we use, as nodes, the vectorized version ($M_{i}\in R^{625}$) of each square in the image, computing the distances between the nodes ($Z\in R^{\left|V|\times |V\right|}$) and apply the prior of smoothness [24]. Lastly, we apply the blue-noise sampling over the smoothed graph $W_{s}$ and inject these samples to the graph based fusion algorithm proposed in [4], in order to obtain the eigenvector with the highest eigenvalue of the fused-graph $W^{f}$. Since the eigenvectors are embedded in a high-dimensional space equal to the resolution of the images (i.e., 960×1280), we use the same dimensionality reduction technique used in [4], based on the *t* distributed stochastic neighbor embedding (*t*-SNE) [36]. Figure 3 summarizes proposed method.

### 3.3. Non-Linear Regression Models

#### 3.3.1. Support Vector Machine Regression (SVM-R)

The purpose of a support vector machine regression is to solve a linear regression problem by mapping the input data into a high dimensional space (feature space). The following model is considered:

(13) $$f\left(w_{e},x\right)=\left(w_{e}*\phi \left(x\right)\right)+b,$$

where $w_{e}$ are the regression coefficients (weights), $\phi \left(x\right):R^{in}\mapsto R^{out}$ with out≫in, and *b* is the bias term. To obtain the optimal values of $W_{e}$, a loss function can be defined such as the Laplacian, Huber’s, Gaussian or ϵ-intensive. We will use the most common loss function that is the ϵ-intensive with a regularization parameter *C*:

(14) $$\begin{array}{cc} \min_{W_{e},\xi ,b} & \;\frac{1}{2}∥W_{e}∥+C\sum_{i=1}^{n}\left(\xi_{i}+\xi_{i}^{*}\right) \end{array}$$

$$\begin{array}{cc} s.t. & \left\{\begin{array}{c} y_{i}-f\left(w_{e},x\right)-b\leq \epsilon +\xi_{i}^{*}\\ f\left(w_{e},x\right)+b-y_{i}\leq \epsilon +\xi_{i}^{*}\\ \xi_{i}^{*},\xi_{i}\geq 0 \end{array}\right., \end{array}$$

where $\xi_{i}^{*}$ and $\xi_{i}$ are slack variables with measure deviations larger than ϵ. By transforming the optimization problem in Equation (14) into a dual problem, the final solution is obtained by using Lagrange, the Karush–Kuhn–Tucker conditions, and the kernel trick [37], giving:

(15) $$f\left(x\right)=\left(\alpha -\alpha^{*}\right)K\left(x\right)+b,$$

where $\alpha ,\alpha^{*}$ are the Lagrange multipliers and K(x) is a Kerrnel function. In this case we use the Radial Basis Function (RBF) Kernel (similar to Equation (7)).

#### 3.3.2. A Nonlinear Autoregressive Exogenous (NARX)

An exogenous autoregressive model (NARX) aimed at the identification of non-linear systems involves both current and past values of the impulsive series that models the dynamics of the system. NARMAX (Nonlinear Autoregressive Moving Average with Exogenous Inputs) methods provide models that are transparent, and easily solve many problems such as contour errors in CNC machines, forecasting, network traffic, and prediction of daily solar radiation [38,39,40,41]. The NARMAX model is defined in Equation (16). Where y(k) is the output system, u(k) is the input system and e(k) is the noise sequence. $n_{y}$, $n_{u}$ y $n_{e}$ are the maximum lags for the system output, input and noise respectively; F[.] refers to the function and *d* is a time delay usually set to d=1.

(16) $$Y\left(k\right)=F\left[y\left(k-1\right),\ldots ,y\left(k-n_{y}\right),u\left(k-d\right),\ldots ,u\left(k-d-n_{u}\right),e\left(k-1\right),\ldots ,e\left(k-n_{e}\right)\right]+e\left(k\right)]$$

In this work, the function F[.] has been implemented using artificial neural networks (ANN) with two hidden layers and one output layer.

## 4. Results and Discussion

### 4.1. Experimental Setup

In order to compare the effectiveness of the proposed feature extraction based on graphs, the results were compared with two approaches: (i) the former graph method introduced in [4], namely GBF, and (ii) the GFKuts approach [3]. We used the datasets and Ground-Truth reported in [3,4], containing 314 images for the vegetation stage, 82 for the reproductive, and 71 for the ripening. The captured images have a resolution of 960×1280 pixels, geo-referenced with the corresponding biomass measurements in grams (*g*) from the Ground-Truth (further information of the experimental protocol can be found at https://www.protocols.io/view/protocol-bjxskpne, accessed date: 24 June 2021), which was assembled as follows: 1 linear meter of the plants were cut from each plot of the crop and weighted to obtain the fresh biomass. Subsequently, the samples are placed inside an oven at 65 degrees Celsius for 4 days or until a constant weight is reached. This is known as the dry biomass.

Having the imagery dataset and the Ground-Truth, we trained two estimation models: classical SVM-R regression and a robust nonlinear autoregressive network with exogenous inputs (NARX), both accounting for 70% of the dataset for the training, and the remaining 30% for testing and validation. Lastly, the performance of the models was measured in terms of the root mean squared error (RMSE), the linear correlation (*r*), and the coefficient of determination ($R^{2}$). Additionally, we conducted several experiments with the SVM regressor considering different kernel functions (i.e., linear, polynomial, and RBF), while the best performance was achieved by the RBF Kernel (similar to the work in [4]).

The algorithms were tested in a server with two Intel(R) Xeon(R) CPUs E5-2650 v4 @ 2.20 GHz, with 24 physical cores, 48 threads of processes, and 252 GB of RAM. Parameters are detailed in Table 1.

### 4.2. Biomass Estimation

**Figure 4:**
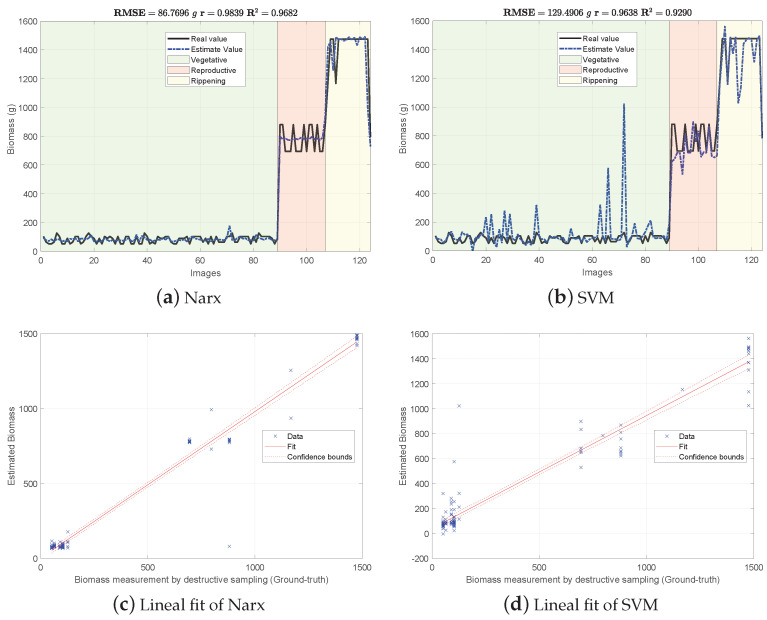
Biomass estimation using the GFKuts approach.

**Figure 5:**
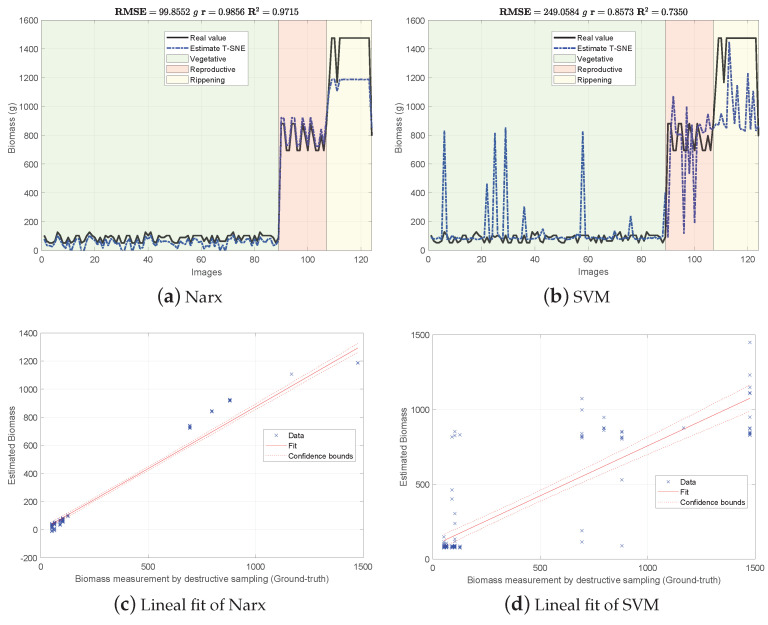
Biomass estimations using the graph-based approach with uniform sampling features (GBF).

**Figure 6:**
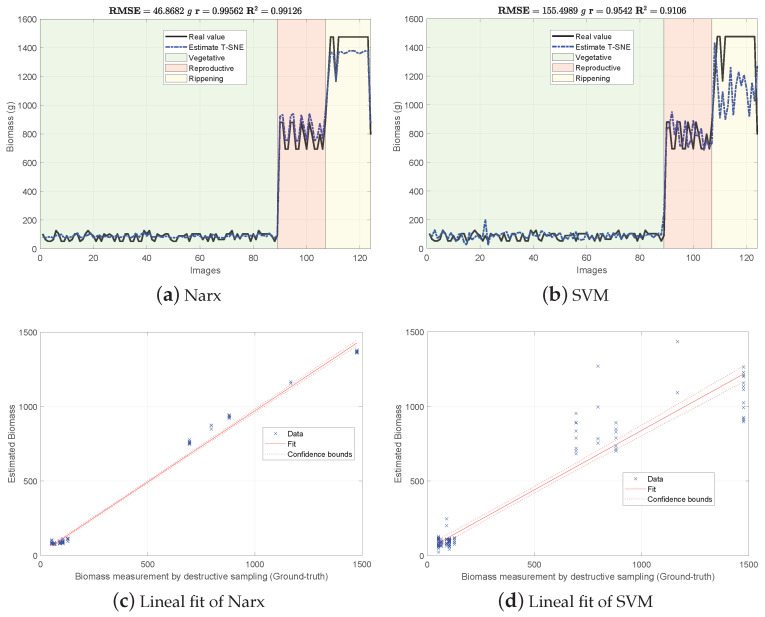
Biomass estimations using the proposed graph-based approach with blue-noise and prior smoothness features (GBF-Sm-Bs).

Figure 4, Figure 5 and Figure 6 show the experimental results obtained from the 3 methods in the following order: (i) GFKuts, (ii) former graph method (GBF), and (iii) proposed graph method with blue-noise and prior smoothness (GBF-Sm-Bs). Numerical data containing the above-ground biomass estimation results for each model are presented in Table 2.

According to Table 2, the GKFkuts approach achieved the lowest RMSE=129.490 g, followed by the proposed graph-based model with RMSE=155.498 g. However, by applying the robust Narx regressor, the best performance was achieved by our proposal, with a RMSE=45.358 g. Results were also correlated to the linear fit, presented from Figure 4, Figure 5 and Figure 6. There we can observe that the Narx regressor improves the fitting to a linear model, in which the proposed method achieved a linear correlation value of r=0.995.

Contrasting with the previous work from [3], the Narx model enabled a slight improvement of about 3% over the former GFKuts method, due mainly to the configuration of the auto regressive inputs, and the use of vegetative indices with the soft mask generated by the GFKuts approach. Regarding the former graph-based method from [4] (GBF), results were improved by around 62.43% (155.498/249.058) and 45.42% (45.358/99.855) for the SVM and Narx regressors in terms of the RMSE metric, respectively. For the SVM regressor, the GBF and the GBF-Sm-Bs methods achieved a RMSE=259.058 g and RMSE=155.498 g respectively, as observed in Table 2, while for the Narx regressor, the GBF and the GBF-Sm-Bs methods achieved a RMSE=99.855 g and RMSE=45.358 g respectively, as presented in Table 2. This demonstrates the effectiveness of using a structured sampling method combined with graph learning, based on a prior of smoothness, for the extraction of relevant samples to be apply into Nyström extension.

Overall, the proposed GBF-Sm-Bs approach using Narx achieved an improvement in the RMSE metric of about 50%, while also improving the biomass correlations reported from previous works. Part of the improvement achieved with the GBF-Sm-Bs method comes from extracting more relevant features than GFKuts, precisely 89 features. While GFKuts works with only 7 VI-features (per image) highly sensitive to biomass variations, the graph-based method is characterized by the eigenvectors with the highest eigenvalue associated with the fused-graph $W^{f}$, yielding 89 features per image.

## 5. Conclusions

The proposed methods for above-ground biomass estimation, not only enabled the precise characterization of the biomass behavior of the rice crops through the entire phenological cycle, but also worked as both image segmentation and feature extraction techniques, by associating relevant features from the canopy. It is worth mentioning that most of the existing body of work in remote sensing methods for high-throughput biomass estimations based on multispectral imagery, requires dedicated photogrammetry methods for image correction, segmentation and feature extraction. In this study, both GFKuts and the GBF-Sm-Bs methods solved those stages by combining them into one single approach.

According to the findings reported in Table 2, the proposed GBF-Sm-Bs approach obtained an average biomass correlation of 0.995 with $R^{2}=0.991$ and RMSE=45.358 g, increasing the precision in the estimation by around 45.42% (45.358/99.855), compared to the GBF method, and about 52.27% (45.358/86.769) compared to the GFKuts approach for the Narx regressor. This is a promising result towards GWAS gene characterization (Genome-wide Association Study), that requires larger amounts of precise and accurate phenotyping data for the association of gene functions with a specific trait. In this regard, future work is aimed at the inclusion of clustering approaches within the proposed algorithms, to enable the extraction of features according to several plant varieties (genotypes).

As future work, it would be interesting to explore the graph convolutional network, which combines the structural information captured by the graphs with the high level of abstraction given by neural networks. Additionally, a strong approach for segmentation (i.e., different for square windows over the image) could lead to improved results, since the segmented regions are used to generated a graph with smoothness prior.

## Figures and Tables

**Table 1:** Parameters used for the models GFKuts [3], GBF [4], and our proposal GBF-Sm-Bs model.

Model	Parameters			
GFKuts [3]	k-neighbors =2	window radius =60	$\epsilon =10^{-6}$	-
GBF [4]	-	-	*t*-SNE${}_{dim}$=16	$n_{s}=100$
GBF-Sm-Bs	edges/node $=\frac{\left|V\right|}{2}$	window size =25	*t*-SNE${}_{dim}$=89	$n_{s}=100$

**Table 2:** Performance in terms of RMSE, *r* and $R^{2}$ for the models: GFKuts [3], GBF [4], and our proposed GBF-Sm-Bs model with both Narx and SVM regressors.

Model/Regressor	RMSE (in grams [g])	*r*	$R^{2}$
GFKuts [3]/SVM	129.490	0.963	0.929
GBF [4]/SVM	249.058	0.857	0.735
GBF-Sm-Bs/SVM	155.498	0.954	0.910
GFKuts [3]/Narx	86.769	0.983	0.968
GBF [4]/Narx	99.855	0.9856	0.971
GBF-Sm-Bs/Narx	45.358	0.995	0.991

## Data Availability

Datasets supporting the experimental results presented in Figure 4, Figure 5 and Figure 6 are available at the Open Science Framework: https://osf.io/cde6h/?view_only=1c4e5e03b9a34d3b96736ad8ab1b2774 folder Raw Data—MDPI Sensors. The experimental protocol for crop monitoring is also available at https://www.protocols.io/view/protocol-bjxskpne (accessed on 24 June 2021).

## References

[1] Ahmed M., Ahmad S., Ahmad S. (2017). Climate Variability Impact on Rice Production: Adaptation and Mitigation Strategies. Quantification of Climate Variability, Adaptation and Mitigation for Agricultural Sustainability.

[2] Alebele Y., Zhang X., Wang W., Yang G., Yao X., Zheng H., Zhu Y., Cao W., Cheng T. (2020). Estimation of Canopy Biomass Components in Paddy Rice from Combined Optical and SAR Data Using Multi-Target Gaussian Regressor Stacking. Remote Sens..

[3] Colorado J.D., Calderon F., Mendez D., Petro E., Rojas J.P., Correa E.S., Mondragon I.F., Rebolledo M.C., Jaramillo-Botero A. (2020). A novel NIR-image segmentation method for the precise estimation of above-ground biomass in rice crops. PLoS ONE.

[4] Jimenez-Sierra D.A., Benítez-Restrepo H.D., Vargas-Cardona H.D., Chanussot J. (2020). Graph-Based Data Fusion Applied to: Change Detection and Biomass Estimation in Rice Crops. Remote Sens..

[5] Yue J., Feng H., Jin X., Yuan H., Li Z., Zhou C., Yang G., Tian Q. (2018). A comparison of crop parameters estimation using images from UAV-mounted snapshot hyperspectral sensor and high-definition digital camera. Remote Sens..

[6] Yue J., Feng H., Yang G., Li Z. (2018). A comparison of regression techniques for estimation of above-ground winter wheat biomass using near-surface spectroscopy. Remote Sens..

[7] Xiao Z., Bao Y.-X., Lin W., Du Z.-Z., Qian T., Can C. (2020). Hyperspectral Features of Rice Canopy and SPAD Values Estimation under the Stress of Rice Leaf Folder. Chin. J. Agrometeorol..

[8] Cheng T., Song R., Li D., Zhou K., Zheng H., Yao X., Tian Y., Cao W., Zhu Y. (2017). Spectroscopic estimation of biomass in canopy components of paddy rice using dry matter and chlorophyll indices. Remote Sens..

[9] Yang X., Jia Z., Yang J., Kasabov N. (2019). Change Detection of Optical Remote Sensing Image Disturbed by Thin Cloud Using Wavelet Coefficient Substitution Algorithm. Sensors.

[10] Li J., Wu Z., Hu Z., Li Z., Wang Y., Molinier M. (2021). Deep Learning Based Thin Cloud Removal Fusing Vegetation Red Edge and Short Wave Infrared Spectral Information for Sentinel-2A Imagery. Remote Sens..

[11] Gitelson A.A., Kaufman Y.J., Stark R., Rundquist D. (2002). Novel algorithms for remote estimation of vegetation fraction. Remote Sens. Environ..

[12] Lin F., Guo S., Tan C., Zhou X., Zhang D. (2020). Identification of Rice Sheath Blight through Spectral Responses Using Hyperspectral Images. Sensors.

[13] Harrell D., Tubana B., Walker T., Phillips S. (2011). Estimating rice grain yield potential using normalized difference vegetation index. Agron. J..

[14] Campos J., García-Ruíz F., Gil E. (2021). Assessment of Vineyard Canopy Characteristics from Vigour Maps Obtained Using UAV and Satellite Imagery. Sensors.

[15] Devia C.A., Rojas J.P., Petro E., Martinez C., Mondragon I.F., Patino D., Rebolledo M.C., Colorado J. (2019). High-throughput biomass estimation in rice crops using UAV multispectral imagery. J. Intell. Robot. Syst..

[16] Colorado J.D., Cera-Bornacelli N., Caldas J.S., Petro E., Rebolledo M.C., Cuellar D., Calderon F., Mondragon I.F., Jaramillo-Botero A. (2020). Estimation of Nitrogen in Rice Crops from UAV-Captured Images. Remote Sens..

[17] Hernández-Vela A., Reyes M., Ponce V., Escalera S. (2012). Grabcut-based human segmentation in video sequences. Sensors.

[18] Rother C., Kolmogorov V., Blake A. (2004). “GrabCut” interactive foreground extraction using iterated graph cuts. ACM Trans. Graph. (TOG).

[19] Mortensen E.N., Barrett W.A. Intelligent scissors for image composition. Proceedings of the 22nd Annual Conference on Computer Graphics and Interactive Techniques.

[20] Xiong J., Po L.M., Cheung K.W., Xian P., Zhao Y., Rehman Y.A.U., Zhang Y. (2021). Edge-Sensitive Left Ventricle Segmentation Using Deep Reinforcement Learning. Sensors.

[21] Liu B., Liu Z., Li Y., Zhang T., Zhang Z. (2021). Iterative Min Cut Clustering Based on Graph Cuts. Sensors.

[22] Boykov Y.Y., Jolly M.P. Interactive graph cuts for optimal boundary & region segmentation of objects in ND images. Proceedings of the Eighth IEEE International Conference on Computer Vision (ICCV 2001).

[23] Castro W., Marcato Junior J., Polidoro C., Osco L.P., Gonçalves W., Rodrigues L., Santos M., Jank L., Barrios S., Valle C. (2020). Deep learning applied to phenotyping of biomass in forages with UAV-based RGB imagery. Sensors.

[24] Kalofolias V., Perraudin N. Large Scale Graph Learning From Smooth Signals. Proceedings of the International Conference on Learning Representations.

[25] Parada-Mayorga A., Lau D.L., Giraldo J.H., Arce G.R. (2019). Blue-noise sampling on graphs. IEEE Trans. Signal Inf. Process. Netw..

[26] Shapiro A. (2003). Monte Carlo sampling methods. Handbooks in Operations Research and Management Science.

[27] Petschnigg G., Szeliski R., Agrawala M., Cohen M., Hoppe H., Toyama K. (2004). Digital photography with flash and no-flash image pairs. ACM Trans. Graph. (TOG).

[28] Correa E.S., Francisco Calderon J.D.C. GFkuts: A novel multispectral image segmentation method applied to precision agriculture. Proceedings of the Virtual Symposium in Plant Omics Sciences (OMICAS).

[29] He K., Sun J., Tang X. (2010). Guided image filtering. Proceedings of the European Conference on Computer Vision.

[30] Fowlkes C., Belongie S., Chung F., Malik J. (2004). Spectral grouping using the Nystrom method. IEEE Trans. Pattern Anal. Mach. Intell..

[31] Kumar S., Mohri M., Talwalkar A. (2012). Sampling methods for the Nyström method. J. Mach. Learn. Res..

[32] Dong X., Thanou D., Rabbat M., Frossard P. (2019). Learning graphs from data: A signal representation perspective. IEEE Signal Process. Mag..

[33] Tanaka Y., Eldar Y.C., Ortega A., Cheung G. (2020). Sampling Signals on Graphs: From Theory to Applications. IEEE Signal Process. Mag..

[34] Iyer G., Chanussot J., Bertozzi A.L. (2020). A Graph-Based Approach for Data Fusion and Segmentation of Multimodal Images. IEEE Trans. Geosci. Remote Sens..

[35] Lau D.L., Arce G.R. (2018). Modern Digital Halftoning.

[36] Van der Maaten L., Hinton G. (2008). Visualizing data using t-SNE. J. Mach. Learn. Res..

[37] Laref R., Losson E., Sava A., Siadat M. (2019). On the optimization of the support vector machine regression hyperparameters setting for gas sensors array applications. Chemom. Intell. Lab. Syst..

[38] Huo F., Poo A.N. (2013). Nonlinear autoregressive network with exogenous inputs based contour error reduction in CNC machines. Int. J. Mach. Tools Manuf..

[39] Men Z., Yee E., Lien F.S., Yang Z., Liu Y. (2014). Ensemble nonlinear autoregressive exogenous artificial neural networks for short-term wind speed and power forecasting. Int. Sch. Res. Not..

[40] Haviluddin, Alfred R. Performance of modeling time series using nonlinear autoregressive with eXogenous input (NARX) in the network traffic forecasting. Proceedings of the 2015 International Conference on Science in Information Technology (ICSITech).

[41] Boussaada Z., Curea O., Remaci A., Camblong H., Mrabet Bellaaj N. (2018). A nonlinear autoregressive exogenous (NARX) neural network model for the prediction of the daily direct solar radiation. Energies.

